# Substitution of the Native Zn(II) with Cd(II), Co(II) and Ni(II) Changes the Downhill Unfolding Mechanism of Ros87 to a Completely Different Scenario

**DOI:** 10.3390/ijms21218285

**Published:** 2020-11-05

**Authors:** Rinaldo Grazioso, Sara García-Viñuales, Luigi Russo, Gianluca D’Abrosca, Sabrina Esposito, Laura Zaccaro, Rosa Iacovino, Danilo Milardi, Roberto Fattorusso, Gaetano Malgieri, Carla Isernia

**Affiliations:** 1 Department of Environmental, Biological and Pharmaceutical Science and Technology, University of Campania—Luigi Vanvitelli, Via Vivaldi 43, 81100 Caserta, Italy; rinaldo.grazioso@unicampania.it (R.G.); luigi.russo2@unicampania.it (L.R.); gianluca.dabrosca@unicampania.it (G.D.); sabrina.esposito@unicampania.it (S.E.); rosa.iacovino@unicampania.it (R.I.); roberto.fattorusso@unicampania.it (R.F.); 2 Institute of Crystallography-CNR, Via Paolo Gaifami 18, 95126 Catania, Italy; saragarvi7@gmail.com (S.G.-V.); dmilardi@unict.it (D.M.); 3 Institute of Biostructures and Bioimaging-CNR (Naples), Via Mezzocannone 16, 80134 Naples, Italy; lzaccaro@unina.it

**Keywords:** cadmium, cobalt, folding mechanism, nickel, zinc finger

## Abstract

The structural effects of zinc replacement by xenobiotic metal ions have been widely studied in several eukaryotic and prokaryotic zinc-finger-containing proteins. The prokaryotic zinc finger, that presents a bigger βββαα domain with a larger hydrophobic core with respect to its eukaryotic counterpart, represents a valuable model protein to study metal ion interaction with metallo-proteins. Several studies have been conducted on Ros87, the DNA binding domain of the prokaryotic zinc finger Ros, and have demonstrated that the domain appears to structurally tolerate Ni(II), albeit with important structural perturbations, but not Pb(II) and Hg(II), and it is in vitro functional when the zinc ion is replaced by Cd(II). We have previously shown that Ros87 unfolding is a two-step process in which a zinc binding intermediate converts to the native structure thorough a delicate downhill folding transition. Here, we explore the folding/unfolding behaviour of Ros87 coordinated to Co(II), Ni(II) or Cd(II), by UV-Vis, CD, DSC and NMR techniques. Interestingly, we show how the substitution of the native metal ion results in complete different folding scenarios. We found a two-state unfolding mechanism for Cd-Ros87 whose metal affinity *K_d_* is comparable to the one obtained for the native Zn-Ros87, and a more complex mechanism for Co-Ros87 and Ni-Ros87, that show higher *K_d_* values. Our data outline the complex cross-correlation between the protein–metal ion equilibrium and the folding mechanism proposing such an interplay as a key factor in the proper metal ion selection by a specific metallo-protein.

## 1. Introduction

Although central in many important biological processes, the mechanisms of metallo-protein folding and assembly are still poorly understood [1]. It is well known that metal ions can have substantial effects on protein stability, folding or unfolding [2,3,4,5,6]. Protein folding is totally metal-dependent in some instances, involving the early coordination of the metal cofactors that, in turn, completely address the folding into the functional native conformation [4,7,8]. On the contrary, metal ions can bind proteins in a later stage of the folding pathway [6]. The relationship of the metal cofactors, protein folding and structural stability can be properly characterized by an integrated approach in which spectroscopic and calorimetric data are combined together [9,10,11,12,13,14]. The study of the influence that different metal ions, with their different electronic structures, have on protein folding/unfolding pathways has been the justification of the present work, intended to contribute to the search for the roles of metal ions in protein folding reactions. The model protein we use is the DNA binding domain of the transcriptional repressor Ros from *Agrobacterium tumefaciens* (named *Ros87*) that has been structurally and functionally characterized in our laboratory [15,16]. Ros87 is the first member of the prokaryotic zinc finger family [17,18,19,20] whose NMR solution structure has been solved [21,22,23,24]. It shows the βββαα globular fold in which the three-dimensional structure is stabilized by a structural zinc ion coordinated by two cysteines (Cys24, Cys27) and two histidines (His37, His42) and by a 15-residue hydrophobic core.

A great number of proteins need metals, such as zinc, copper, iron, cobalt, nickel, manganese, magnesium, and calcium. Proteins are known to bind copper and zinc strongly, but bind metals such as manganese, magnesium and calcium less tightly [25]. Some non-essential metals, such as cadmium and mercury, can also be highly competitive. In particular, the substitution of the native zinc with cadmium within the coordination complex of zinc-finger motifs [26,27] has been proposed as a possible mechanism through which this xenobiotic metal may exert its toxicity.

Cells are known to limit the amounts of metal ions available within the cytoplasm while providing sufficient amounts of each metal to the different proteins. It is recognised that each protein competes with other proteins for a limited pool of metals.

It is well known how toxicological exposure to metals or a breakdown in the homeostatic mechanism that regulates the amount of metals present in a cell results in abnormal cellular mechanisms [28]. In these conditions, proteins interact with concentrations of metal ions different from the physiological conditions and this interaction often results in protein inactivation, misfolding and aggregation. Misfolding and aggregation are at the basis of a lot of diseases and there are a plethora of data indicating that metal ions are capable of accelerating these processes.

In this scenario, we have previously shown that, although with different affinities, Ros87 is able to fold when the native structural zinc ion is substituted by cadmium, cobalt and nickel [29,30,31]. In the present work, we investigate whether this structural tolerance and the differences in binding affinities for the different metals shown by Ros87 result in alterations in its folding mechanism. Protein fold characterization plays a crucial role in uncovering the three-dimensional structure of proteins and protein functions as nascent folding intermediates can play an important role in disease pathogenesis [32].

Our spectroscopic and calorimetric data are integrated to establish the existence and the magnitude of folding energy barriers and provide information on temperature-dependent conformational changes in Ros87 upon zinc substitution.

## 2. Results

### 2.1. Co(II)—Ros87 Thermal Unfolding

**Figure 1:**
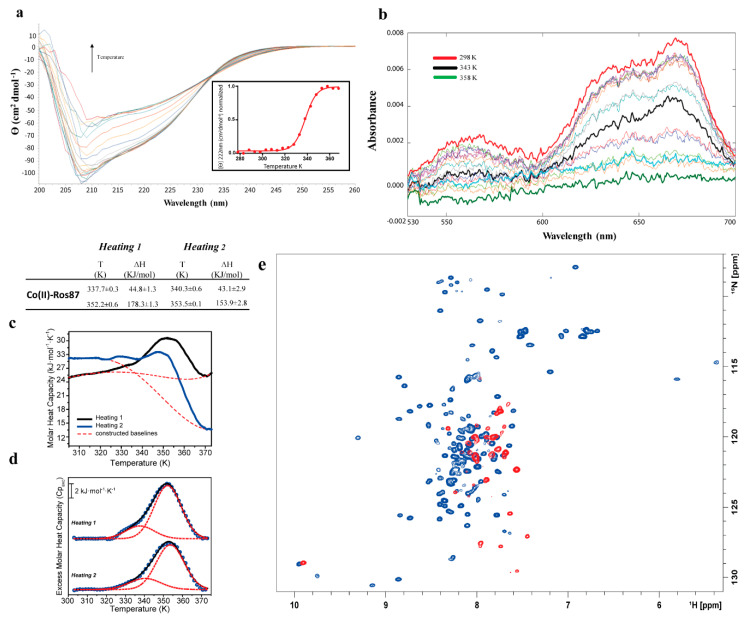
Thermal unfolding of Co(II)-Ros87. (**a**) CD spectra recorded between 298–368 K; the inset shows the melting curve followed at 222 nm fitted with a sigmoidal curve ([Co(II)-Ros87] = 10 µM, pH = 6.5). (**b**) Portion of the UV-Vis spectra at different temperatures (298–358 K) ([Co(II)-Ros87] = 15 µM, pH = 6.5) (**c**,**d**) DSC thermal unfolding curves and fitting of two different heating cycles ([Co(II)-Ros87] = ~100 µM, pH = 6.5). (**e**) Overlay of the ^1^H-^15^N HSQC spectra of Co(II)-Ros87 at 298 K (blue) and 343 K (red) ([Co(II)-Ros87] = ~250 µM, pH = 6.5).

Due to its electronic configuration Co(II) is normally used to characterize zinc binding to zinc finger motifs. In fact, cobalt and zinc have nearly the same ionic radius (0.58 and 0.60 Å, respectively), and in all the cases the apparent *K_d_* of the zinc–protein complex is obtained with an indirect approach by monitoring the cobalt displacement [30]. Given the importance of the cobalt as a spectroscopic probe [33], we started to study the effect of cobalt to zinc substitution on the thermal unfolding of Ros87. Figure 1a reports CD spectra recorded as a function of temperature: data show how the raise in temperature results in a progressive loss of secondary structure content. The θ value at 222 nm was followed, obtaining a melting temperature (Tm) of 338 ± 1 K.

**Figure 2:**
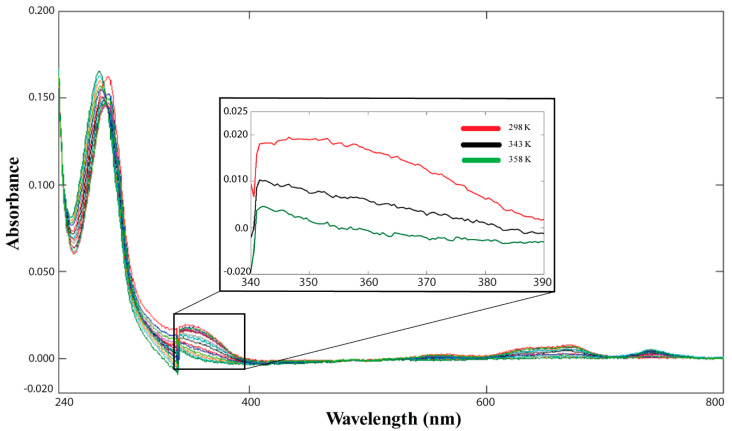
UV-Vis unfolding of Co(II)-Ros87 ([Co(II)-Ros87] = 15 µM, pH = 6.5); the inset reports an enlargement for three representative temperatures of the spectral region encompassing 340–390 nm.

Co(II)-Ros87 thermal unfolding was also followed via UV-Vis spectroscopy and a reduction in the absorbance was observed as function of temperature (Figure 1b). In particular, we closely examined the absorption bands between 340–390 nm, which are commonly used to evaluate the number of cysteines involved in Co(II) coordination [30] (Figure 2). 

The UV-Vis spectrum recorded at 298 K gives a ε value of 2500 M^−1^ cm^−1^, consistent with the involvement of both cysteines in the tetrahedral coordination of the Co(II) ion [30]. Interestingly, the calculated epsilon value decreases as a function of temperature with a complete lowering of the band at 358 K (ε = 133 M^−1^ cm^−1^), halving its value at 343 K (ε = 1333 M^−1^ cm^−1^). The comparison of the epsilon estimated at this temperature with the value obtained at 298 K indicates that only half of the cysteines contained in our sample coordinate the metal ion. 

Co(II)-Ros87 thermograms recorded via DSC (Figure 1c,d) show two different endothermic transitions: a first broad transition spanning 25 K (from 322 to 357 K) centered at Tm = 337.7 ± 0.3 K and a second broad transition from 333 to 370 K with a Tm = 352.2 ± 0.6 K. According to the second heating–cooling cycle, the first transition is a reversible process, associated with a change in enthalpy of about 45 kJ/mol. In an attempt to elucidate the mechanism of folding, this transition has been fitted with a two-state model. However, the calculated calorimetric to van’t Hoff enthalpy ratio (r = ΔH_VH_/ΔH) was far from unity, and this allow us to exclude a two-state folding mechanism for Co(II)-Ros87 [34]. The second transition involves an enthalpy change of about 178 kJ/mol and is partially reversible (78%).

In order to describe the unfolding mechanism of Co(II)-Ros87 at atomic resolution, we acquired a series of ^1^H-^15^N HSQC spectra as function of the temperature between 298 and 343 K at intervals of 5 K (Figure 1e). In particular, the comparison of the NMR spectra (Figure S1) show that most of the residues disappear at 338 K and reappear at 343 K with a tight distribution of signals indicating that the protein is almost completely disordered. This behaviour indicates that the first transition is characterized by a cooperative thermal unfolding in which the folded Co(II)-Ros87 conformational exchanges with the apo-Ros87 in the micro-millisecond timescale.

**Figure 3:**
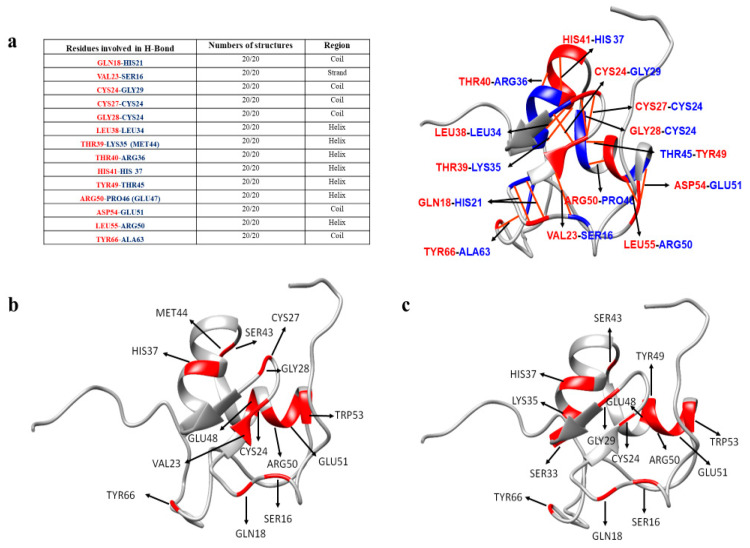
Intramolecular Zn(II)-Ros87 hydrogen bonds. (**a**) Hydrogen bonds present in all the 20 structures of Ros87 NMR ensemble (PDB code 2JSP): in red are highlighted the donor and in blue the acceptor. (**b**) Ni(II)-Ros87 residues involved in intramolecular H-bonds shown onto the NMR structure of Ros87. (**c**) Cd(II)-Ros87 residues involved in intramolecular H-bonds shown onto the NMR structure of Ros87.

To better understand how the cobalt to zinc substitution affect the thermal unfolding of Ros87 we investigated the structural and dynamical proprieties of Co(II)-Ros87 at 298 K by applying an alternative strategy in which we evaluated the H_N_ chemical shift variations as function of temperature [35] without performing the time-consuming, sequence-specific assignment procedure. In particular, we measured the amide-proton temperature coefficients ΔδH_N_/ΔT (ppb/K) for 43 residues that are well resolved in the ^1^H-^15^N HSQC spectrum at 298 K, and then we compared the obtained data with the Zn(II)-Ros87 NMR structure (PDB ID code: 2JSP). Amide-proton temperature coefficients are related to the presence of intramolecular H-bonds and a positive linear coefficient indicates a simple thermal expansion process, whereas a positive but non-linear coefficient can mean that a bond is not only getting longer but is also sampling an alternative conformation. As rule, values more negative than −4.6 ppb/K are commonly observed in protein–solvent hydrogen bonds, while temperature coefficients less negative than −4.6 ppb/K tend to be associated with intra-molecular bonds, frequently observed in secondary structure contacts. We found 14 residues for which the amide proton is involved in a strong hydrogen bond (ΔδH_N_ > −4.6 ppb/K), whereas 16 out of a total of 43 residues show temperature coefficients smaller than −4.6 ppb/K, indicating the presence of solvent-exposed protons (Table S1). In addition, the measured amide temperature coefficients show 13 residues characterized by weak hydrogen bonding or a mixture of hydrogen-bonded and solvent-exposed amide protons. Notably, the number of strong hydrogen bonds identified by our strategy is in excellent agreement with that obtained by analyzing the 20 conformers of the Zn(II)-Ros87 NMR ensemble (Figure 3a). Interestingly, most of the residues (~63%) with ΔδH_N_ > −4.6 ppb/K show non-linear shifts with a temperature increase, suggesting that Ros87, upon cobalt binding, samples alternative conformations. Overall, our analysis indicates that Co(II)-Ros87, despite having a similar secondary and tertiary organization of the zinc-loaded form, presents a more complex conformational equilibrium. 

When the samples are cooled back to 298 K, the CD, NMR and UV-Vis experiments are in agreement to show that the protein is able to return to bind the metal and to refold, as shown in Supporting Information (Figure S2). It should be here noted that, because of instrumental limitations, the samples were heated to 343 K in the NMR experiments, while it was possible to reach 368 K in the CD and UV-Vis experiments. 

### 2.2. Ni(II)-Ros87 Thermal Unfolding 

**Figure 4:**
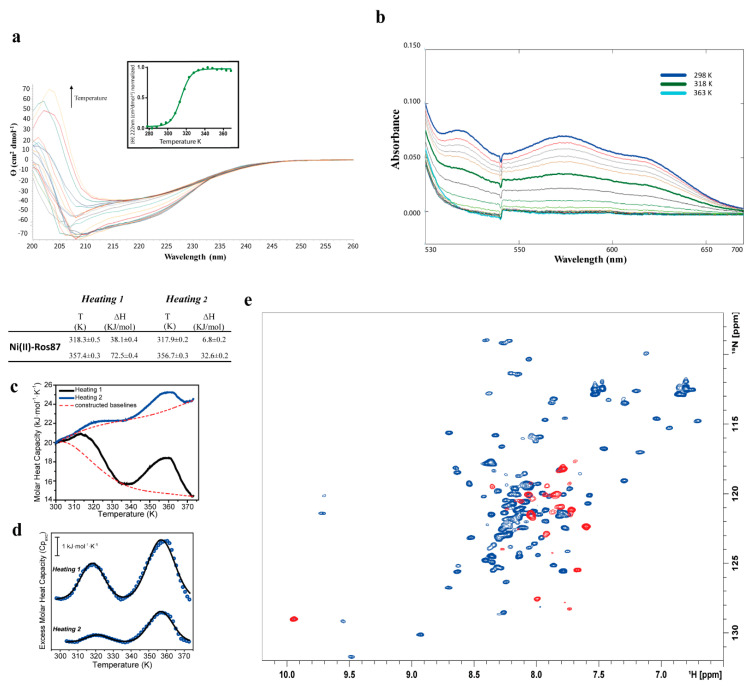
Thermal unfolding of Ni(II)-Ros87. (**a**) CD spectra recorded between 298–368 K; the inset shows the melting curve followed at 222 nm fitted with a sigmoidal curve ([Ni(II)-Ros87] = 10 µM, pH = 6.5). (**b**) Portion of the UV-Vis spectra at different temperatures (298–368 K) ([Ni(II)-Ros87] = 40 µM, pH = 6.5) (**c**,**d**) DSC thermal unfolding curves and fitting of two different heating cycles ([Ni(II)-Ros87] = ~100 µM, pH = 6.5). (**e**) Overlay of the ^1^H-^15^N HSQC spectra of Ni(II)-Ros87 acquired at 298 K (blue) and 343 K (red) ([Ni(II)-Ros87] = ~250 µM, pH = 6.5).

Figure 4a reports CD spectra of Ni(II)-Ros87 [30] recorded as a function of temperature. As already observed for Co(II)-Ros87, Ni(II)-Ros87 data show how the rise in temperature results in a progressive loss of secondary structure content. The Ɵ value at 222 nm was followed and data exhibit a sigmoidal unfolding transition centered at approximately 314 ± 0.7 K, which is sensitively lower when compared to the value found for Co(II)-Ros87. These data reflect the different affinities measured for the two metal ion complexes in this buffer. (K_d_* Ni(II) = (2.3 (±0.3) × 10^−6^ M, K_d_* Co(II) = (5.59 (±1.97) × 10^−8^).

Ni(II)-Ros87 thermal unfolding was also followed via UV-Vis spectroscopy (Figure 4b) and also, in this case, a reduction in the absorbance was observed as a function of temperature, obtaining a mid-point transition temperature of 317 ± 1.3 K, in a substantial agreement with the CD data. Interestingly, the final UV-Vis spectrum recorded when the sample is cooled back to 298 K shows that the protein is no longer able to coordinate the Ni(II) ion (Figure S3). 

DSC experiments were also carried out to study the thermal profile of the protein complex (Figure 4c,d). The obtained thermograms showed an endothermic transition from 293 to 330 K, centered about Tm = 318.3 ± 0.5 K with and associated enthalpy change of about 38 kJ/mol, in agreement with CD and UV-Vis data. A re-heating run showed again the transition observed for the first heating cycle, albeit with a significantly smaller contribution of about 7 kJ/mol. This indicates that the endothermic transition is mostly irreversible, showing only about 18% of refolding, consistently with what is observed with the above-mentioned spectroscopic techniques. As occurred with Co(II)-Ros87, the calorimetric to van’t Hoff enthalpy ratio is far from unified, allowing to exclude a two-state folding mechanism. A second endothermic transition was also observed in the temperature range from 338 to 373 K centered at about Tm = 357.4 ± 0.3 K. This transition was partially reversible (45%) with enthalpic changes of about 72 and 33 kJ/mol during the first and the second heating, respectively. This enthalpic change, however, cannot not be directly associated with changes in the tertiary or secondary structure of the protein, since at 318 K the protein is already unfolded.

In the case of Ni(II)-Ros87, the ^1^H-^15^N HSQC spectra (Figure 4e and Figure S4) recorded in the range 298 to 343 K confirm the behavior observed using the other two spectroscopic techniques: a loss of the tertiary and secondary interactions is already evident at 318 K where the spectrum demonstrates the presence in the solution of a completely unfolded polypeptide. In agreement with the irreversible behavior observed via CD and UV-Vis, cooling down the sample to 298 K points out that the protein is not able to restore the intra-molecular interactions necessary to obtain a well folded protein (Figure S3).

In the case of Ni(II)-Ros87, we have characterized the first transition following in the ^1^H-^15^N HSQCs the chemical shift variation of 49 well resolved signals as function of temperature. In the case of Ni(II)-Ros87, we previously assigned the backbone of the protein [31] and documented the structural perturbations due to the substitution of Zn(II) with Ni(II), a smaller ionic radius metal. For this reason, a residue by residue detailed analysis was possible. Out of the 49 investigated signals, 14 residues (see Figure 3b and Table S1) show a temperature coefficient constantly > −4.6 ppb/K within the investigated temperature range, indicating the involvement of these aminoacids in stable H-bonds. Ten of them, however, show conformational exchanges after 308 K, as indicated by the slight slope change of the fitting curve observed at this temperature. A total of 31 residues are not involved in H-bonds, 12 of which show non-linear ^1^H shifts with temperature in the ^1^H-^15^N HSQC experiments. A total of 14 residues have temperature coefficient > −4.6 ppb/K up to 323 K and < −4.6 ppb/K after 323 K, indicating their involvement in H-bonds that are lost with the temperature rise. Consistently with the slight structural perturbations already documented [31], most of the residues originally found in Zn(II)-Ros87 to be involved in H-bonds are conserved in Ni(II)-Ros87. However, Leu38, Thr39 and Thr40 (involved in the formation of H-bonds in Zn(II)-Ros87 and belonging to the first α-helix) are not involved in the formation of H-bonds in Ni(II)-Ros87. Two extra residues in Ni(II)-Ros87 show the involvement in stable H-bonds: Ser43 and Met44 localized in the turn between the two helices and Glu48 and Trp53 in the second α-helix.

### 2.3. Cd(II)—Ros87 Thermal Unfolding

**Figure 5:**
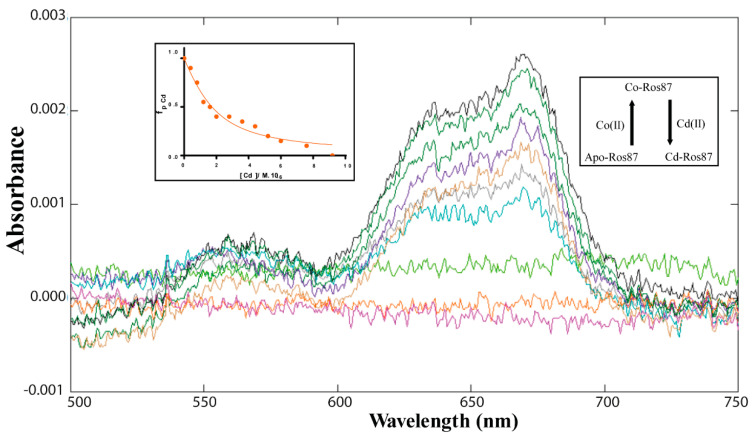
Ros87-Cd(II) binding affinity. Portion of the UV-Vis spectra of Co(II)-Ros87 titration with CdCl_2_ ([Co(II)-Ros87] = 10 µM). The inset shows the absorbance values at 670 nm fitted with the binding isotherm reported in the Experimental Section.

The study of Cd(II)-Ros87 started with the determination of Ros87 affinity for Cd(II). We have previously reported the affinity constant for this metal–protein interaction, but the data were collected in phosphate buffer (pH = 6.8) [29]. The magnitude of dissociation constants is largely dependent upon the buffer in which the characterization is performed. For this reason, in order to obtain data comparable with those collected for the Co(II) and Ni(II) complexes, the Cd(II) apparent dissociation constant for Ros87 has been re-determined (Figure 5), as reported in the materials and methods section, obtaining K_d_* = (9.7 (±1) × 10^−9^).

**Figure 6:**
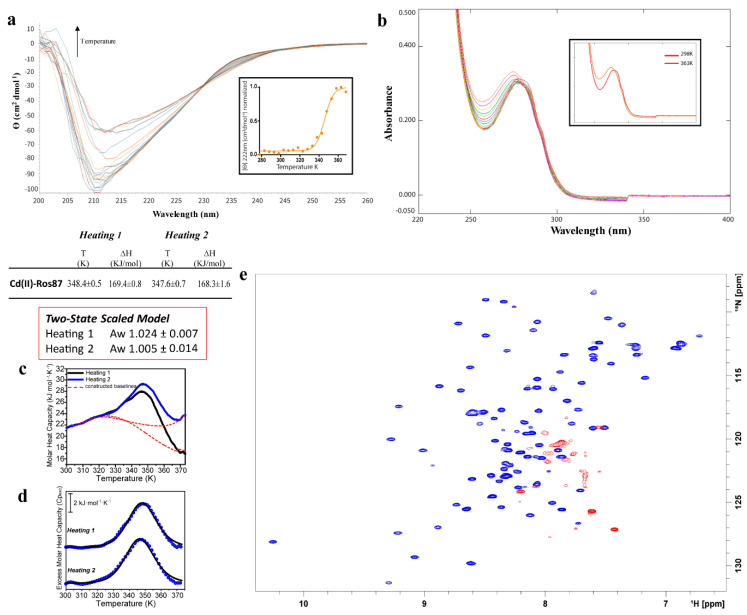
Thermal unfolding of Cd(II)-Ros87. (**a**) CD spectra recorded between 298–368 K the inset shows the melting curve followed at 222 nm fitted with a sigmoidal curve ([Cd(II)-Ros87] = 15 µM, pH = 6.5). (**b**) Portion of the UV-vis spectra at different temperatures (298–368 K) ([Cd(II)-Ros87] = 26 µM, pH = 6.5) (**c**,**d**) DSC thermal unfolding curves and fitting of two different heating cycles ([Cd(II)-Ros87] = ~100 µM, pH = 6.5). (**e**) Overlay of the ^1^H-^15^N HSQC spectra of Cd(II)-Ros87 at 298 K (blue) and 343 K (red) ([Cd(II)-Ros87] = ~250 µM, pH = 6.5).

Figure 6a reports the CD spectra of Cd(II)-Ros87 recorded as a function of temperature, also showing in this case a progressive loss of secondary structure. Fitting the Ɵ value at 222 nm using a two-state model, a Tm of 345 ± 1.4 K is obtained. In this case, thermal unfolding was followed via UV-Vis spectroscopy (Figure 6b) but, unlike the other two cases, no reduction in the absorbance was observed.

In particular, the spectra at the highest temperature show an intense LMCT absorption band (due to S^¯^ → Cd(II) ligand to metal charge transfer) in the near UV, indicative of the involvement of the cysteine residues in the metal coordination [27]. These finds are further supported by the analysis of the ^13^C_β_ NMR chemical shifts in Cys24 and Cys27 at 343 K (Table 1) that are typical of cysteines involved in metal ion coordination [36].

The ^1^H-^15^N HSQC spectrum of Cd(II)-Ros87 and its superposition with the same spectrum of Zn(II)-Ros87 indicate that the protein is able to accommodate the larger Cd(II) ion within its coordination sphere with only minor structural rearrangements. These data are in agreement with the data reported in the phosphate buffer. The NMR melting experiments (Figure 6e and Figure S5) recorded in the range from 298 to 343 K confirm the behavior observed using the other two spectroscopic techniques: Cd(II)-Ros87 unfolds according to a two-state mechanism. In fact, the pattern of signals in the spectrum recorded at 333 K is consistent with the presence of a folded peptide; all the resonances disappear at 343 K. Differently from what was previously observed in the presence of Zn(II), this analysis outlines that no intermediate species for Cd(II)-Ros87 were sampled during the explored temperature range.

Figure 6c, d shows the DSC thermogram of Cd(II)-Ros87, in which significant differences with Co(II)-Ros87 and Ni(II)-Ros87 thermograms can be readily observed. The Cd(II)-Ros87 thermogram presents only one endothermic transition centred at about 348.4 ± 0.5 K. The transition is totally reversible, showing an enthalpic change of about 170 kJ/mol in both heating cycles. Differently from what was observed with Ros87 complexes with Co(II) and Ni(II), Cp_exc_(T) traces (Figure 6c) fit perfectly with a two-state model with an average van’t Hoff to calorimetric enthalpy ratio (ΔH_vH_/ΔH_cal_) of 1.024 (±0.007), supporting the above-mentioned spectroscopic findings, which indicate the lack of intermediate species during the transition. 

In the case of Cd(II)-Ros87, we analyzed the chemical shift variation of 63 well-resolved signals as a function of temperature (Table S2). In the case of Cd(II)-Ros87, the structural perturbations due to the substitution of Zn(II) with Cd(II), a larger ionic radius metal, have been documented [29]. A Cd(II)-Ros87 backbone was assigned. Out of the 63 investigated signals, 14 residues (Table S2) show an amide proton temperature coefficient that indicates the involvement of these amino acids in stable H-bonds within the entire investigated temperature range. Seven of them, however, show a change in their dynamics in the milliseconds time scale after 323 K, as indicated by the slight slope change in the fitting curve observed at this temperature. A total of 33 residues are not involved in H-bonds, 14 of which show a change in their dynamics. A total of 16 residues have a temperature coefficient indicating their involvement in H-bonds that are lost when the temperature rises to 323 K. Consistently with the slight structural perturbations already documented [29], most of the residues originally found in Zn(II)-Ros87 to be involved in H-bonds are conserved in Cd(II)-Ros87. Differently from what was found for Zn(II)-Ros87, Leu38 and Thr40 are not involved in the formation of H-bonds in Cd(II)-Ros87, while Ser33, Ser43, Glu48 and Trp53 are involved in H-bonds.

The unfolding of Cd(II)-Ros87 is clearly reversible, as indicated by all the used spectroscopic techniques (Figure S6). 

## 3. Discussion

Metal coordination is necessary for the function of a lot of proteins; however, metal ions are not only an important element of structures, but they are also known to play major role in their stability and dynamics [37,38]. The biosynthesis of proteins coordinating a metal opens up a series of important questions: does the metal ion bind before, during or after the folding process of the synthesized protein? After the metal is bound, how does its coordination influence the stability and dynamics of a protein? How does a protein select the proper metal ion?

The comprehension of how metals are used by proteins in cells on a molecular level requires accurate descriptions of the thermodynamic and kinetic parameters involved in protein–metal complexes [37]. Biophysical studies of how interactions with a certain metal ion affect the folding, stability and conformational dynamics of metal-binding proteins are an important complement to structural data and in vivo assays. Despite a wealth of structural data having been collected in the last decades, there has been much less focus on the folding processes of metal-binding proteins. 

It is clear that a metal ion is able to bind specifically to an unfolded or partially folded structure in vitro, and thereby affect its folding processes. It is also clear that the roles of metal ions in protein stability and folding in vitro can vary dramatically.

Here, we investigate, by means of an integrated approach that combines calorimetric and spectroscopic data, the folding behavior of the protein Ros87, a prototype of the prokaryotic Ros/MucR zinc finger family [17,39] that represents a valuable paradigm to study the influences of different metal ions on protein folding scenarios [40] (Table 2).

In previous works, we have demonstrated that proteins belonging to this family can overcome the structural metal requirement to achieve the proper functional fold: in some Ros homologues that bear a different combination of residues substituting the metal-coordinating residues, a network of H-bonds and hydrophobic interactions surrogate the structure stabilizing role played by the metal cofactor [41]. In these proteins, the same structure of Ros87 is achieved in the absence of the structural Zn(II) [39]. We have also shown how this striking difference between members of the same protein family resulted in different mechanism of folding to achieve the same globular βββαα architecture. In particular, we have demonstrated that the metal-free homologue, Ml4_52–151_, folds in the structure that characterizes the prokaryotic zinc finger family via a classic two-state cooperative transition while the metal-binding Ros87 exhibits a complex folding scenario presenting a well-defined metal-binding intermediate that converts in the native state through a delicate barrier-less downhill folding mechanism. The metal-binding intermediate is constituted by the turn connecting the two coordinating cysteines, the second coordinating histidine and the second α-helix. We have also previously demonstrated how the substitution of the native metal zinc with Cd(II), Ni(II) and Co(II) results in a well folded protein with small structural rearrangements [29,30,31].

In the present work, we demonstrate how these metal substitutions, according to our previous structural data, although allowing the protein to retain the same globular architecture, result in a destabilization of the first α-helix that bears the two coordinating histidines with a loss of key H-bonds and a gain in H-bonds that characterize the second helix and the turn connecting the two helices. More importantly, these alterations result in differences in Ros87 mechanisms of folding. In detail, Cd(II)-Ros87, reflecting the *K_d_* very similar to that of Zn(II)-Ros87, appears much more stable than Co(II)-Ros87 and Ni(II)-Ros87; it folds using a classical two-state mechanism. DSC shows the single broad transition that typifies this mechanism of folding. Accordingly, a CD reversible thermal unfolding profile can be fitted to a classical two-state model with a melting temperature of 345 ± 1.4 K, and the disappearance during thermal unfolding of the ^1^H-^15^N resonances around Tm is in agreement with a two-state unfolding mechanism. This behavior is consistent with a folding/unfolding conformational exchange in the µs-to-ms timescale. Interestingly, the carbon chemical shift and the UV-vis data demonstrate that, at the highest measured temperature, the metal ion is still bound to the two coordinating cysteines. However, the largest ionic radius of the cadmium ion bound by the two cysteines with respect to that of the zinc ion does not seem to favor the formation of a stable intermediate. Analogously, in the case of Ni(II) and Co(II), it appears evident that their smaller ionic radii, with respect to that of the native zinc, render the two metal-protein complexes less stable. 

Co(II)-Ros87 unfolding is more complicated, being characterized by two transitions in the DCS thermogram: the first transition between 322 to 357 K and the second from 333 to 370 K. The first transition, in agreement with the chemical shift behavior in the same temperature range, presents a melting temperature of ~338 K. Accordingly, CD reversible thermal unfolding exhibits an unfolding transition at a Tm of 338 ± 1 K, 9 K below that of Cd(II)-Ros87. The UV-Vis data demonstrate that, at 343 K, 50% of Co(II)-Ros87 is in conformational equilibrium with the free form of the protein while, at 358 K, the end of the first DSC transition, there is a complete loss of the metal cofactor. The second transition is only partially reversible (78%) and is likely due to final loss of the residual structures, the formation of aberrant disulfide bonds involving the two free Cys residues and a dynamic equilibrium between aggregated proto-structures [42]. Ni(II) is bound to Ros87 with the highest *K_d_*, and the unfolding behavior of Ni(II)-Ros87 is different from that of the other two metal–protein complexes. Ni(II)-Ros87 DSC shows a first irreversible transition (only ~18% of refolding) and a second partially reversible transition. CD, UV-Vis and NMR document a loss of the tertiary and secondary interactions at ~318 K, where the NMR spectrum demonstrates the presence in the solution of a completely unfolded polypeptide. We can speculate that the loss of the metal ion at such a low temperature leads, along with the temperature rise, to the early formation of aggregated structures, possibly due to the oxidation of cysteines that do not dis-aggregate by lowering the temperature. This event leads to the second transition that is only partially similar to the one observed in the case of Co(II)-Ros87, with only 45% of reversibility. Free cysteines are commonly observed to form aberrant disulfide bonds in aggregates, and they have been proposed to also play elusive roles in modifying noncovalent interactions during aggregation [42]. 

## 4. Experimental Section

### 4.1. Protein Expression and Purification

^15^N labelled or un-labelled proteins employed for the UV-Vis, CD, NMR and DSC experiments were over-expressed and purified, as earlier reported [16]. 

Briefly, the plasmid was introduced into *E. coli* host strain BL21(DE3) and, for selection, transformed bacteria were plated onto an LB-agar plate containing ampicillin (100 µg/mL). 

^15^N labeling was obtained by growing the cells at 37 °C in a minimal medium that contained ^15^NH_4_Cl as only nitrogen source. At OD_600_ = ~0.6, the expression was induced for 1 h 30 min with 1.0 mM isopropyl-β-D-thiogalactopyranoside (IPTG). Cells were harvested by centrifugation (3750 rpm for 40 min) and the pellet was resuspended in 20 mM Na_2_HPO_4_ (pH 6.8) buffer. The suspension was lysed by sonication and centrifuged at 16,500 rpm for 40 min. The supernatant was filtrated with a 0.22 µm filter membrane to remove cell debris and applied to a Mono S HR 5/5 cation exchange chromatography column (Amersham Bioscences, Amersham, UK) equilibrated with phosphate buffer. The fractions with the proteins were collected and applied to a HiLoad 26/60 Superdex 75 (Amersham Bioscences, Amersham, UK) gel filtration chromatography column equilibrated with 20 mM Na_2_HPO_4_ (pH 6.8), 0.2 M NaCl.

Amicon ultra-15 (Merck, Burlington, MA, USA) centrifugal filter was used to concentrate proteins after the purification phase to reach the desired final concentration. The zinc ion was removed from the samples by acidifying native Zn(II)-Ros87 at pH ~2.5 adding HCl 0.1 M and dialyzing it against a 10 mM Tris, 150 µM TCEP (400 µM for NMR experiments) aqueous solution at pH 2.5. The pH was fixed to 6.5 and controlled throughout the experiments.

### 4.2. UV-Vis Spectroscopy 

UV–Vis spectra were recorded at room temperature in 10 mM Tris, 150 µM TCEP at pH 6.5, on Shimadzu UV-1800 spectrophotometer (Kyoto, Japan) in the range 200–800 nm.

Apo-Ros87 concentration was obtained by determining the absorbance at 280 nm at pH 2.5 using a molar absorption coefficient of 9970 M^−1^ cm^−1^ (www.expasy.org). 

In the case of cadmium [17,43], to obtain a binding isotherm rather than a saturation curve (obtained with the direct titration), and to estimate the Cd(II) binding affinity, a reverse titration of Co(II)-Ros87 with CdCl_2_ (5 mM) was performed up to a Cd(II):Co(II)-Ros87 ratio of 2.4:1. Co(II)-Ros87 binding complexation was followed by direct titration of the apo-protein solution (10 µM) with CoCl_2_ solution (5 mM) up to 1.5 Co(II):protein ratio. Titration of Co(II)-Ros87 with Cadmium induces a decrease in the absorption band at 670 nm. To estimate the dissociation constant of Cd(II)-Ros87 complex, the fractional saturation values were calculated. The data obtained were fitted by the following binding isotherm

$$\begin{array}{c} f_{pCd}=\frac{A_{x}-A_{max}}{A_{0}-A_{max}}\;=\\ \frac{(K_{d}^{Co}{\left[P\right]}_{tot}+K_{d}^{Co}{\left[Cd\right]}_{tot}+K_{d}^{Cd}{\left[Co\right]}_{tot}-K_{d}^{Cd}{\left[P\right]}_{tot}\;\sqrt{{\left(K_{d}^{Co}{\left[P\right]}_{tot}+K_{d}^{Co}{\left[Cd\right]}_{tot}+K_{d}^{Cd}{\left[Co\right]}_{tot}-K_{d}^{Cd}{\left[P\right]}_{tot}\right)}^{2}-4{\left[P\right]}_{tot}\left(K_{d}^{Co}-K_{d}^{Cd}\right)K_{d}^{Co}{\left[Cd\right]}_{tot}}}{2{\left[P\right]}_{tot}\left(K_{d}^{Co}-K_{d}^{Cd}\right)} \end{array}$$

where *f_pCd_* is the fractional saturation; *A*_0_ and *A_max_* are the absorbance values in the absence and presence of Me(II), respectively; [*Cd*]*_tot_* is the total Cadmium concentration; [*Co*]*_tot_* is the total concentration of cobalt added; *K_d_^Cd^* and *K_d_^Co^* are the apparent dissociation constants of the Cd(II)-protein complex and Co(II)-protein complex, respectively. The fitting of the data gave, in all cases, a good R-square. Data were fitted using the program GraphPad Prism 7.0.

UV–Vis spectra of Co(II)-Ros87, Ni(II)-Ros87 and Cd(II)-Ros87 for thermal unfolding experiments were recorded at increasing temperature from 298 to 363 K in 10 mM Tris, 150 µM TCEP at pH 6.5, on Shimadzu UV-1800 spectrophotometer (Kyoto, Japan) equipped with a Peltier temperature control, in the range 200–800 nm. CoCl_2_, NiCl_2_ and CdCl_2_ stock solutions (5 mM) were added to the protein sample to reach a final metal:protein ratio of 2.4:1.

### 4.3. CD Spectroscopy 

Thermal denaturation of Ros87 proteins complexed to the different metals (Co(II), Ni(II) and Cd(II)) was performed using a JASCOJ-815 CD spectropolarimeter (Tokyo, Japan) equipped with Peltier temperature control. Protein samples were prepared in 10 mM Tris, 150 µM TCEP adjusted at pH 6.5. CD spectra were measured at 5 K intervals in the temperature range of 278–373 K. At the end, the samples were cooled back to 298 K and a final set of spectra were collected. The experiments were conducted on ~10/15 µM of Ros87 samples and fresh solutions of 5.0 mM MeCl_2_ were used to fully load the apo-proteins up to a final [Me(II)]:[protein] ratio of 2.4:1.

Data were collected using a quartz cuvette with a 1 cm path-length in the 200−260 nm wavelength range with a data pitch of 1 nm. To remove the background contribution of buffer, all data were recorded with a bandwidth of 1 nm with a scanning speed of 50 nm/min and normalized against reference spectra. 

### 4.4. NMR Spectroscopy 

NMR samples were made of ~250 μM solution of ^15^N Ros87, ~600 μM Me(II) (metal:protein = 2.4:1), 10 mM Tris, and 400 μM TCEP adjusted to pH 6.5 in 550 μL of 90% H_2_O/10% ^2^H_2_O. The NMR spectra were recorded on a Bruker Avance III HD 600 MHz (Billerica, MA, USA), equipped with a cryoprobe, at the Department of Environmental, Biological and Pharmaceutical Science and Technology, University of Campania “L. Vanvitelli” (Caserta, Italy). For the thermal unfolding experiments, a series of ^1^H−^15^N HSQC spectra were acquired increasing temperatures at regular intervals of 5 K from 298 to 343 K with the following parameters: the number of complex points was 256 for ^15^N (F1), 1024 for ^1^H (F2). Resonance assignments for backbone NH, ^15^N, Cα, as well as Cβ nuclei were obtained for Cd(II)-Ros87 using inter-residue connectivities detected in triple-resonance HNCA [44] and CACB(CO)NH [44] experiments. Data were processed using TopSpin 3.5 (Bruker, Billerica, MA, USA) and NMRPIPE [45] software and analyzed with CARA [46] and SPARKY [47] software.

### 4.5. Differential Scanning Calorimetry (DSC)

DSC experiments were performed using a NanoDSC instrument (TA Instruments, New Castle, DE, USA). Protein solutions were prepared after extensive dialysis against the buffer (10 mM Tris, 150 µM TCEP, pH 6.5), then, complexes were prepared by adding the different metals ions (Co(II), Ni(II) and Cd(II)) to the previously prepared protein solution in a metal/protein molar ratio 2.4:1. All protein samples, after a vacuum degassing process, were heated at 1 K/min in the temperature range 278−373 K. An extra external nitrogen pressure of about 3 atm was applied to the solution to prevent the formation of air bubbles during heating. In all measurements, the buffer from the last dialysis step, after adding the corresponding metal (in a molar concentration 1.4-fold the protein amount), was used in the reference cell of the calorimeter. To ensure a proper equilibration of the calorimeter, several buffer−buffer heating scans were routinely performed prior to the measurement. To obtain the molar heat capacity curves Cp(T), buffer−buffer/metal baselines were recorded at the same scanning rate and then subtracted from raw DSC curves and normalized by the protein concentration. In all experiments, two heating−cooling cycles were carried out to determine the reversibility of the process. Excess molar heat capacities curves (Cp_exc_) were obtained from Cp(T), by subtracting a baseline obtained by a fifth-order polynomial fit of the pre- and post-transition Cp trends, as described elsewhere [48]. The number of DSC components to be adopted in the peak deconvolution procedure was selected in order to minimize fitting errors. Cp_exc_ curves were deconvoluted by the NanoAnalyze software using the Gaussians model, except in case of Cd(II)-Ros87 in which the Two-State scaled model was used. The temperatures (Tm) and enthalpy (ΔH) of protein melting are defined as the temperature at which the Cp_exc_ curve reaches its maximum value and the area under the Cp_exc_(T) peak, respectively.

## 5. Conclusions

In order to comprehend biological function and/or failure of a protein on a molecular level, it is extremely important to define not only the structure but also the folding pathways, together with thermodynamic and kinetic aspects of reaching the functional structure.

It is well known that there is an interplay in vivo between folding and metal acquisition for metallo-proteins. The important question seems to be at what point in the folding pathway a given protein interacts with the proper metal ion. Some metals are delivered to the proper protein by pathways involving metallo-chaperones, in which case the specific protein–protein interaction can guarantee that only the correct proteins acquire the metal [25].

However, it has been suggested that the distribution of metal ions within the cellular compartment in which the protein folding happens could control metal binding specificity in proteins with binding sites appropriate for more than one metal kind [37]. Moreover, temperature changes or pH could impact proper protein folding, leading to an uptake of different available metals. 

Heavy metals and metalloids in vitro can hinder the correct folding of denatured proteins while, in living cells, they might hamper nascent proteins folding and cause their aggregation [49,50,51]. While folding experiments in vitro are commonly executed with purified proteins in the most favorable conditions for refolding, in vivo folding occurs in a crowded environment that renders the intracellular pathway of protein folding less simple. However, in both situations, the same unique three-dimensional structure of a given protein is achieved [52]. Both in vitro or in vivo proteins are susceptible to metals during folding because the unique three-dimensional structure is stabilized by the same interactions and, in both situations, a given protein is likely to sample the same folding intermediate states in which the different amino acid side chains are solvent exposed [52,53].

Altogether, our in vitro study reports evidences pinpointing that different metal ions, with different K_d_s, different ionic radii and different electronic structures, are surely able to stabilize the three-dimensional structure of our proposed model protein (Ros87), but also participate in and are determinant of its folding pathway. The native Zn(II) ion confers to the functional Ros87 its partly downhill folding mechanism [10]. It is noted that many proteins involved in degenerative disorders have metal-binding capabilities [54,55,56,57]. For this reason, the here-demonstrated influence on Ros87 mechanism of folding of different metals suggests a possible mechanism through which concentrations of metal ions different from the physiological conditions may exert their toxicity in vivo. 

The here-reported data outline how different metal ions interplay with the protein backbone conformation and may result in diverse folding/unfolding mechanisms. As metal ion substitution with Co(II) or Ni(II) is commonly used in bioinorganic studies [58,59,60], especially in classical reverse-titration methods used to estimate the affinities of Zn(II) for zinc binding sites (in particular zinc fingers), our results outline a key point to keep in consideration when exploring metallo-protein features: these two metal ions, because of their different ionic radii and different electronic features, have different thermodynamic effects on the protein structure that result in a differentiation of the stability of the metal complexes.

## Figures and Tables

**Table 1:** Cβ Chemical shift in Cysteine residues at different temperatures.

	Chemical Shift at 298 K (ppm)	Chemical Shift at 308 K (ppm)	Chemical Shift at 328 K (ppm)	Chemical Shift at 343 K (ppm)
Cβ Cys 24	32.63 (±0.4)	33.05 (±0.4)	33.24 (±0.4)	33.12 (±0.4)
Cβ Cys 27	33.68 (±0.4)	33.08 (±0.4)	33.79 (±0.4)	33.64 (±0.4)

Reference values: Cβ oxidized: 41.17 (±3.93) ppm. Cβ reduced: 28.92 (±2.11) ppm. Cβ Metal coordination 30.89 (±1.01) ppm.

**Table 2:** Summary of Tm (K) and ΔH (kJ/mol) values obtained for the Ros87 metal complexes.

	Tm (K)	ΔH (kJ/mol)
Co(II)-Ros87	CD: 338 ± 1	
	UV-Vis: 341 ± 2	
	DSC 1st transition 1st Cycle: 337.7 ± 0.3	1st Cycle: 44.8 ± 1.3
	DSC 1st transition 2nd Cycle: 340.3 ± 0.6	2nd Cycle: 43.1 ± 2.9
	DSC 2nd transition 1st Cycle: 352.2 ± 0.6	1st Cycle: 178.3 ± 1.3
	DSC 2nd transition 2nd Cycle: 353.5± 0.1	2nd Cycle: 153.9 ± 2.8
	NMR: 338	
Ni(II)-Ros87	CD: 314 ± 0.7	
	UV-Vis: 317 ± 1.3	
	DSC 1st transition 1st Cycle: 318.3 ± 0.5	1st Cycle: 38.1 ± 0.4
	DSC 1st transition 2nd Cycle: 317.9 ± 0.2	2nd Cycle: 6.7 ± 0.2
	DSC 2nd transition 1st Cycle: 357.4 ± 0.3	1st Cycle: 72.5 ± 0.4
	DSC 2nd transition 2nd Cycle: 356.7 ± 0.3	2nd Cycle: 32.6 ± 0.2
	NMR: 318	
Cd(II)-Ros87	CD: 345 ± 1.4	
	UV-Vis: None	
	DSC 1st transition 1st Cycle: 348.4 ± 0.5	1st Cycle: 169.4 ± 0.8
	DSC 1st transition 2nd Cycle: 347.6 ± 0.7	2nd Cycle: 168.3 ± 1.6
	NMR: 343	

## Supplementary Materials

The following are available online at https://www.mdpi.com/1422-0067/21/21/8285/s1, Table S1: Temperature coefficients (ppb/K) of Ni(II)-Ros87; Table S2: Temperature coefficients (ppb/K) of Cd(II)-Ros87; Figure S1: Thermal unfolding of Co(II)-Ros87 via NMR; Figure S2: Reversibility of the thermal unfolding of Co(II)-Ros87; Figure S3: Reversibility of the thermal unfolding of Ni(II)-Ros87; Figure S4: Thermal unfolding of Ni(II)-Ros87 via NMR; Figure S5: Thermal unfolding of Cd(II)-Ros87 via NMR; Figure S6: Reversibility of the thermal unfolding of Cd(II)-Ros87.
