# Supplementary material for: Substitution of the Native Zn(II) with Cd(II), Co(II) and Ni(II) Changes the Downhill Unfolding Mechanism of Ros87 to a Completely Different Scenario
Source: Int J Mol Sci. 2020 Nov 5;21(21):8285. doi: 10.3390/ijms21218285 (PMC7663847; doi:10.3390/ijms21218285)
Supplement: Supplementary file 1 [file ijms-21-08285-s001.pdf]

SUPPORTING INFORMATION

for

Substitution of the native Zn(II) with Cd(II), Co(II) and Ni(II) changes the downhill unfolding mechanism of Ros87 to a completely different scenario

Rinaldo Grazioso[1], Sara García-Viñuales[2], Luigi Russo[1], Gianluca D'Abrosca[1], Sabrina Esposito[1], Laura Zaccaro[3], Rosa Iacovino[1], Danilo Milardi[2], Roberto Fattorusso[1], Gaetano Malgieri[1*] and Carla Isernia[1*]

1: Department of Environmental, Biological and Pharmaceutical Science and Technology – University of Campania - Luigi Vanvitelli, via Vivaldi 43, 81100 Caserta (Italy).

2: Institute of Crystallography-CNR, Via Paolo Gaifami 18, 95126 Catania (Italy).

3: Institute of Biostructures and Bioimaging-CNR (Naples), Via Mezzocannone 16, 80134 Naples, Italy

*to whom correspondence should be addressed

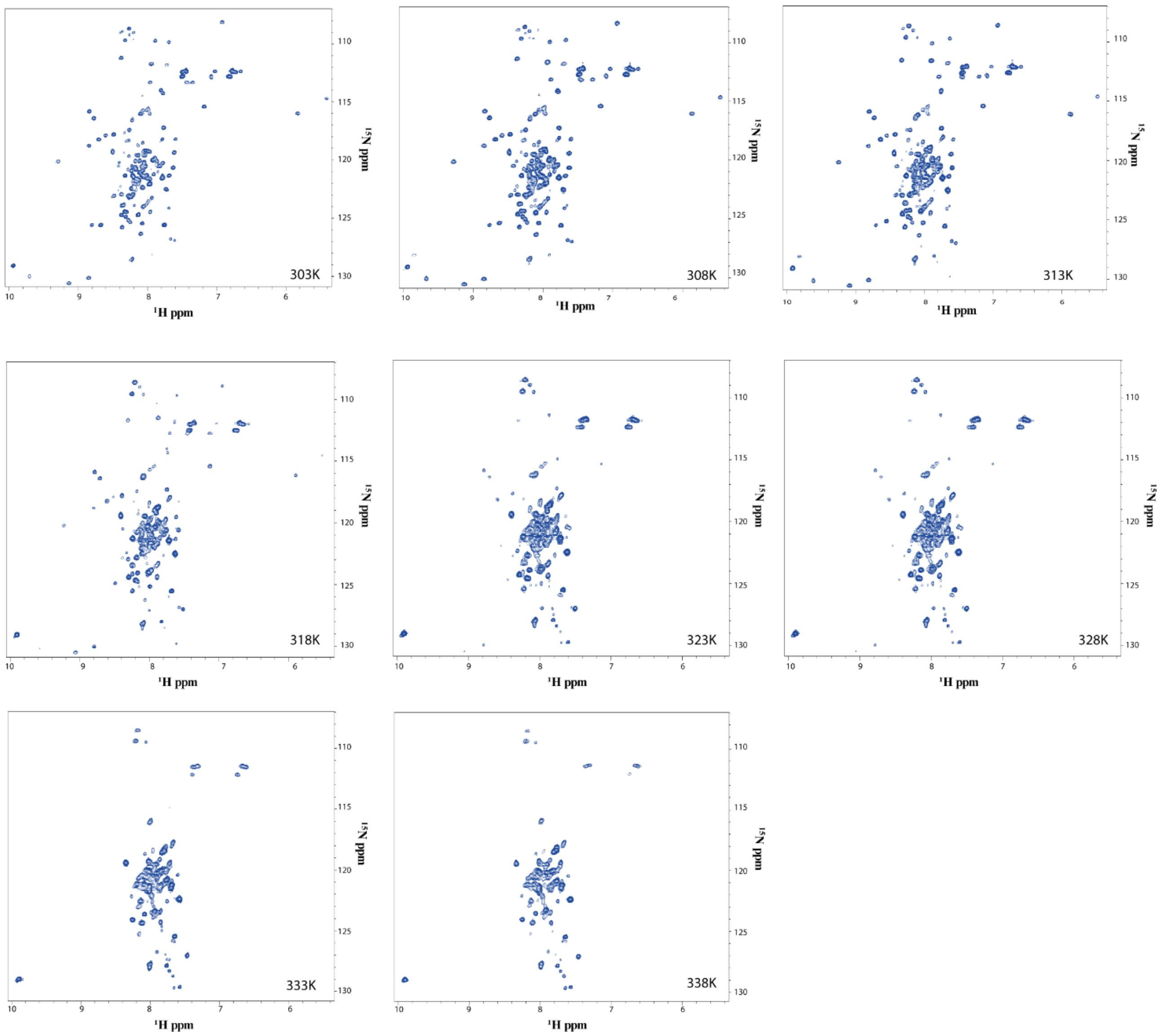

Figure S1. Thermal unfolding of Co(II)-Ros87 via NMR: ^{1}H-^{15}N HSQC spectra acquired at different temperatures using a 600 MHz spectrometer.

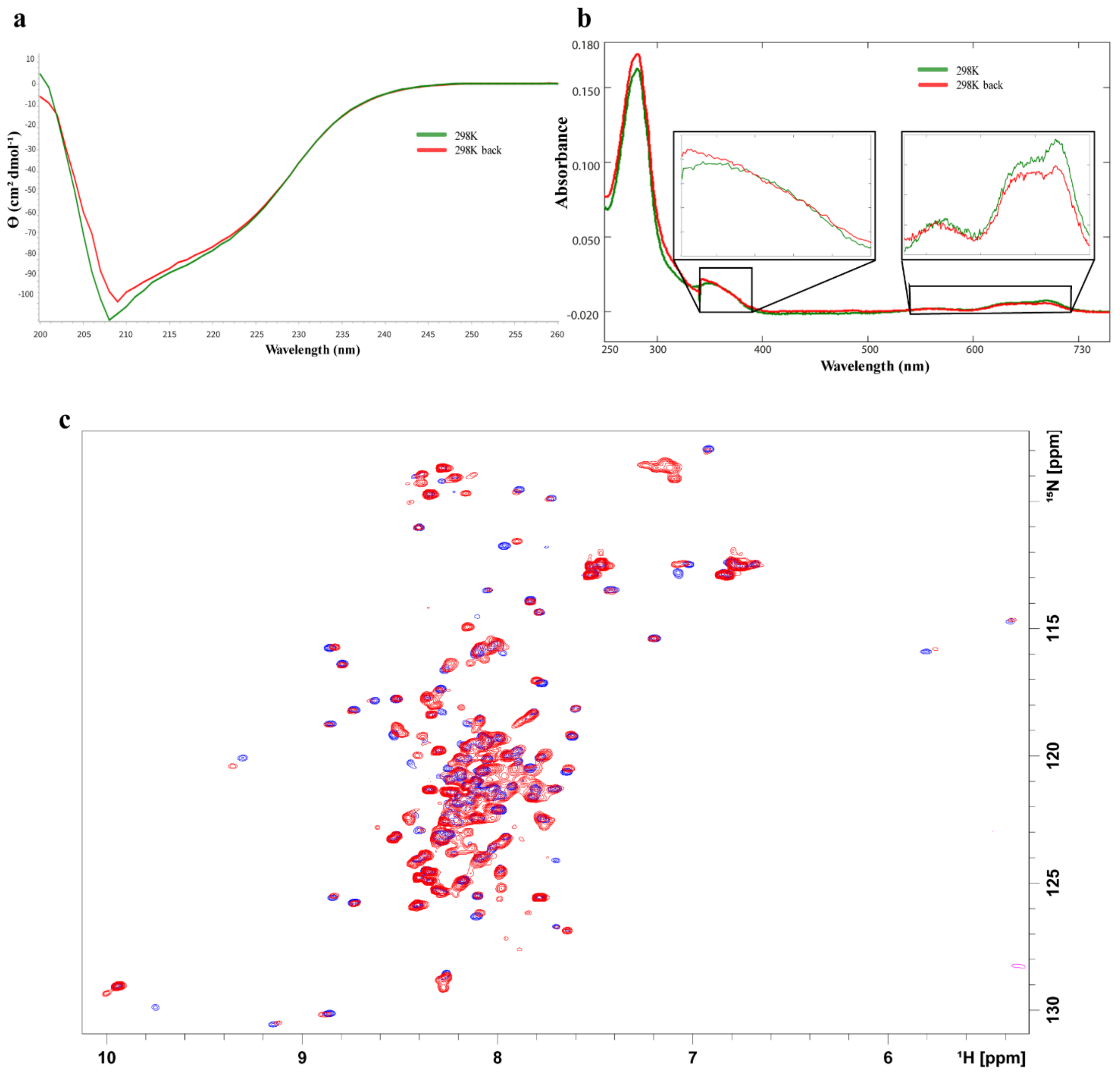

Figure S2. Reversibility of the thermal unfolding of Co(II)-Ros87. **a)** CD spectra acquired at 298 K before (green) and after heating the sample at 368 K (red). **b)** UV-Vis spectra at 298 K before (green) and after heating the sample at 368 K (red); the most significant regions of the spectrum are magnified (350-400nm and 550-690nm). **c)** superposition of the ^{1}H-^{15}N HSQC spectra of Co(II)-Ros87 acquired at 298 K before (blue) and after heating the sample at 343 K (red), respectively.

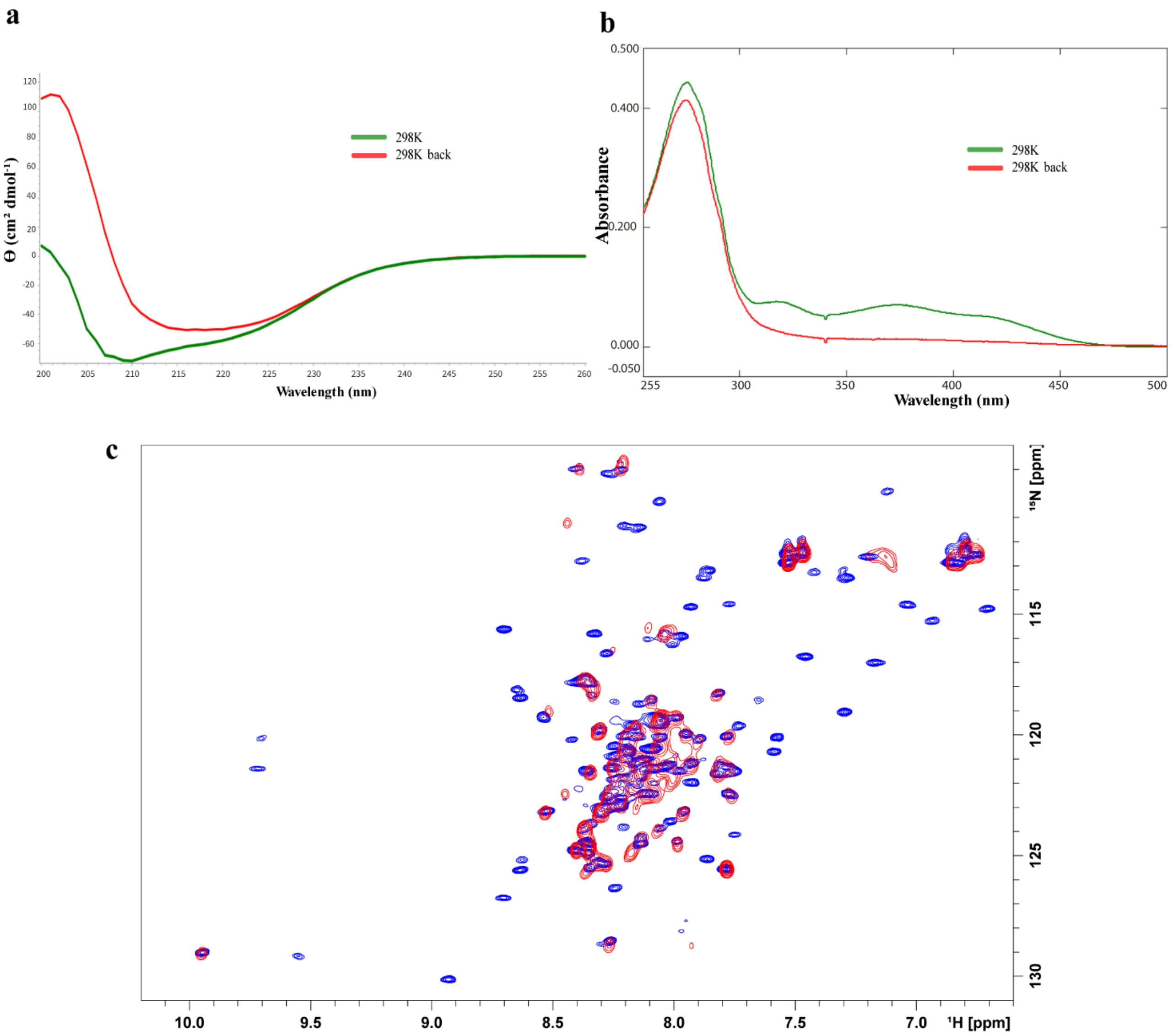

Figure S3. Reversibility of the thermal unfolding of Ni(II)-Ros87. **a)** CD spectra acquired at 298 K before (green) and after heating the sample to 368 K (red), respectively. **b)** UV-Vis spectra at 298 K before (green) and after heating the sample at 368 K (red). **c)** superposition of the ^{1}H-^{15}N HSQC spectra of Ni(II)-Ros87 recorded at 298K before (blue) and after heating the sample at 343K (red).

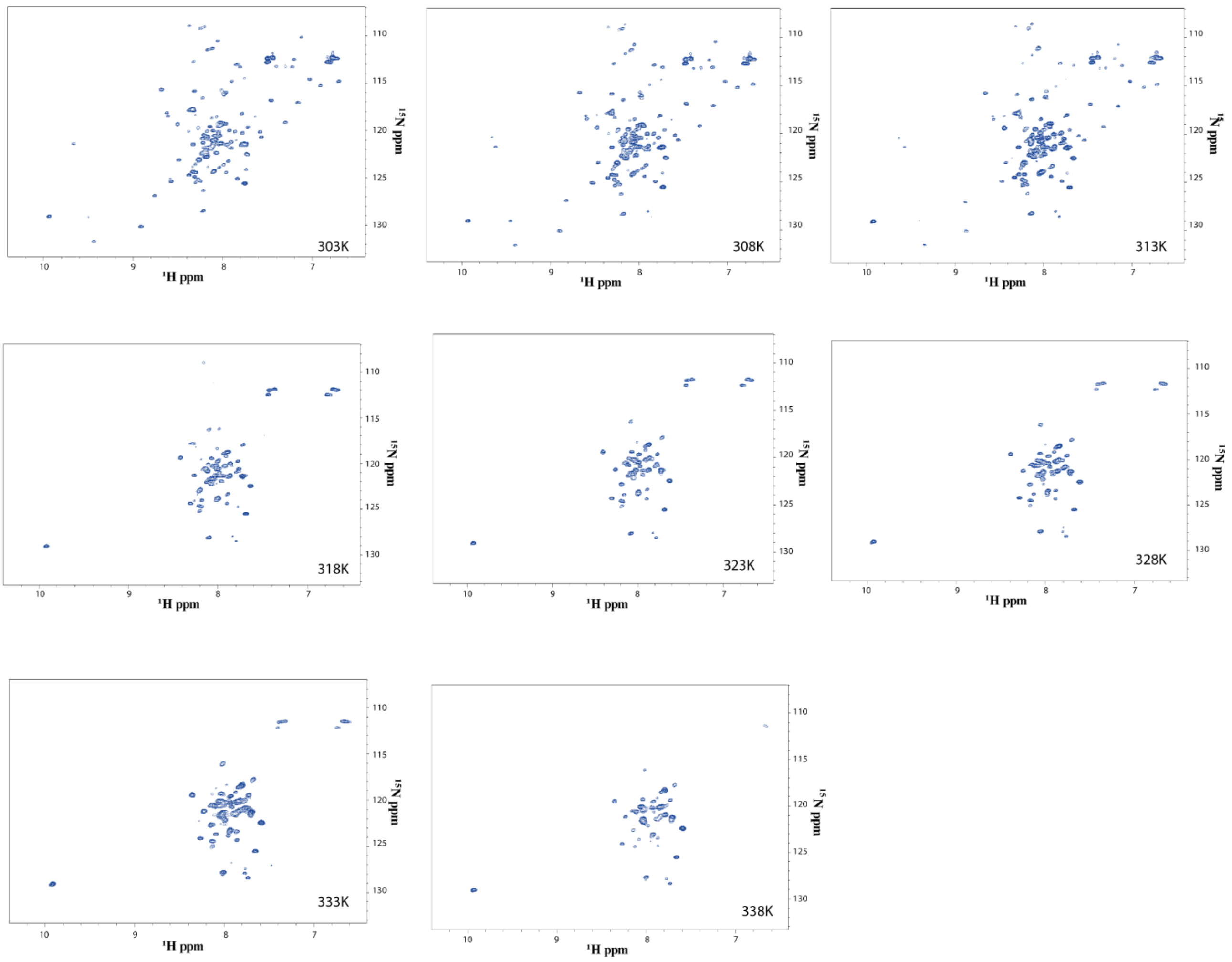

Figure S4. Thermal unfolding of Ni(II)-Ros87 monitored via NMR: ^{1}H-^{15}N HSQC spectra measured at different temperatures.

ΔδHN/ΔT **(ppb/K)**	**Residues with non linear coefficients**	**Residues with linear coefficients**
>-4.6	SER16<br>VAL 23<br>CYS24<br>GLY28<br>HIS37<br>SER43<br>MET44<br>GLU48<br>ARG50<br>TYR66	GLN18<br>CYS27<br>GLU51<br>TRP53
<-4.6	ASP20<br>LEU25<br>SER30<br>LEU34<br>LYS35<br>LEU38<br>HIS42<br>ALA69<br>GLY78<br>GLY80<br>ARG82<br>LYS84	GLU5<br>LYS6<br>GLN7<br>SER12<br>VAL13<br>ASP19<br>HIS21<br>GLY29<br>PHE31<br>ARG36<br>THR39<br>THR40<br>HIS41<br>THR45<br>ASP58<br>MET77<br>GLN87<br>ALA85<br>ARG87
**>-4.6 from 298 K to 323 K and <-4.6 from 323K to 338K**	VAL4<br>LYS8<br>ALA10<br>VAL17<br>GLU26<br>GLU47<br>LYS55<br>TYR59<br>ALA65<br>ALA68<br>SER71<br>ALA74<br>LYS75<br>LEU79	

Table S1. Amide-proton temperature coefficients (ppb/K) of Ni(II)-Ros87.

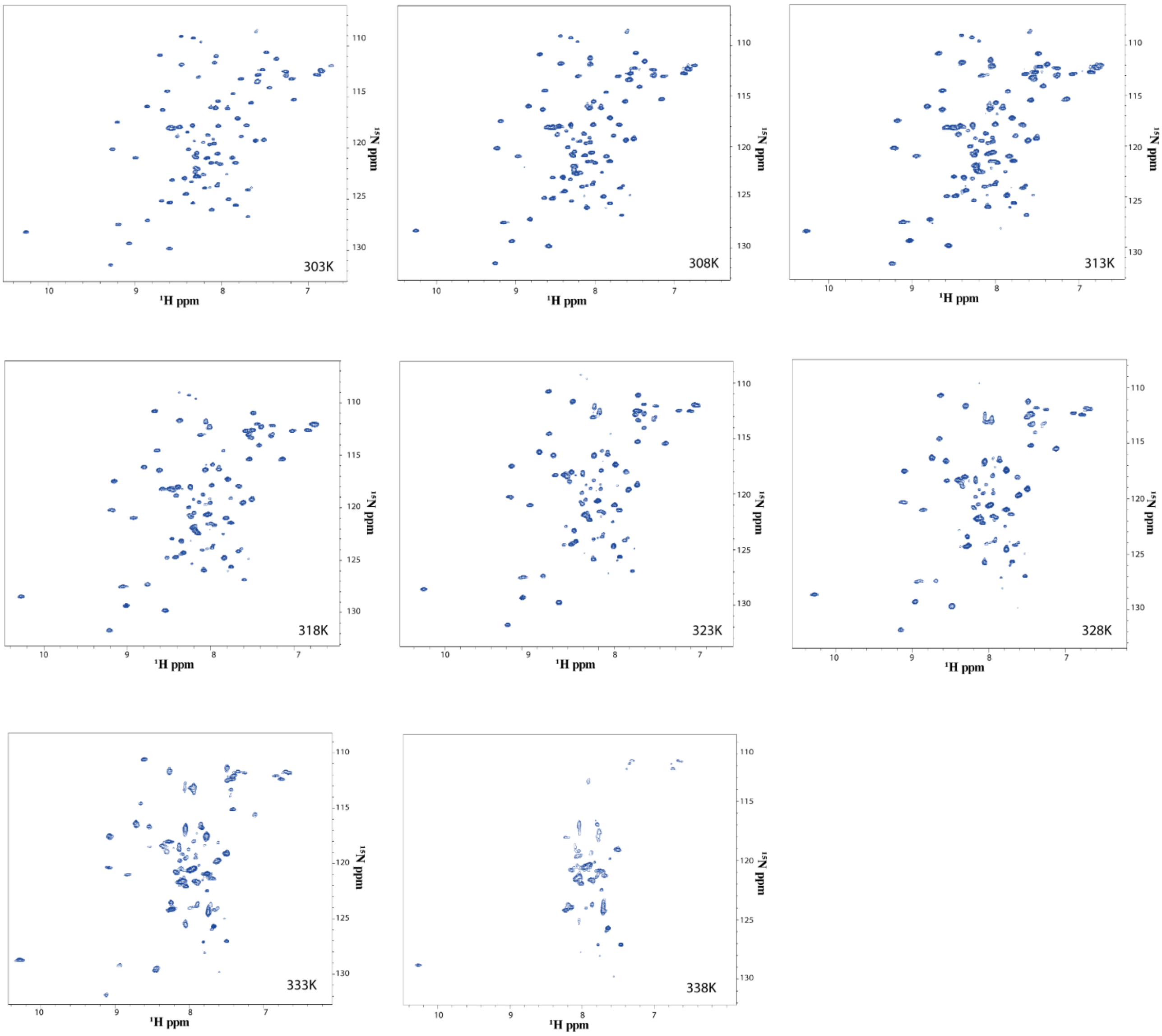

Figure S5. Thermal unfolding of Cd(II)-Ros87 investigated by NMR: ^{1}H-^{15}N HSQC spectra collected at different temperatures.

$\Delta\delta HN/\Delta T$ **(ppb/K)**	**Residues with non linear coefficients**	**Residues with linear coefficients**
>-4.6	SER16<br>CYS24<br>GLY29<br>SER33<br>HIS 37<br>ARG50<br>GLU51	GLN18<br>LYS35<br>SER43<br>GLU48<br>TYR49<br>TRP53<br>TYR66
<-4.6	ALA10<br>VAL13<br>ASP19<br>ILE22<br>VAL23<br>CYS27<br>LEU38<br>THR45<br>LEU55<br>VAL57<br>TYR59<br>GLU68<br>GLN81<br>ARG87	VAL4<br>GLU5<br>GLN7<br>VAL17<br>HIS21<br>SER30<br>PHE31<br>LEU34<br>ARG36<br>THR39<br>THR40<br>HIS41<br>ALA65<br>ALA74<br>LYS75<br>MET77<br>GLY80<br>ARG82<br>ALA85
**>-4.6 from 298 K to 323 K and <-4.6 from 323K to 338K**	LYS6<br>SER12<br>LEU25<br>GLU26<br>MET44<br>GLU47<br>LYS52<br>ASP58<br>ALA63<br>ALA69<br>ARG70<br>GLU76<br>GLY78<br>LEU79	MET61<br>LEU73

Table S2. Amide-proton temperature coefficients (ppb/K) of Cd(II)-Ros87.

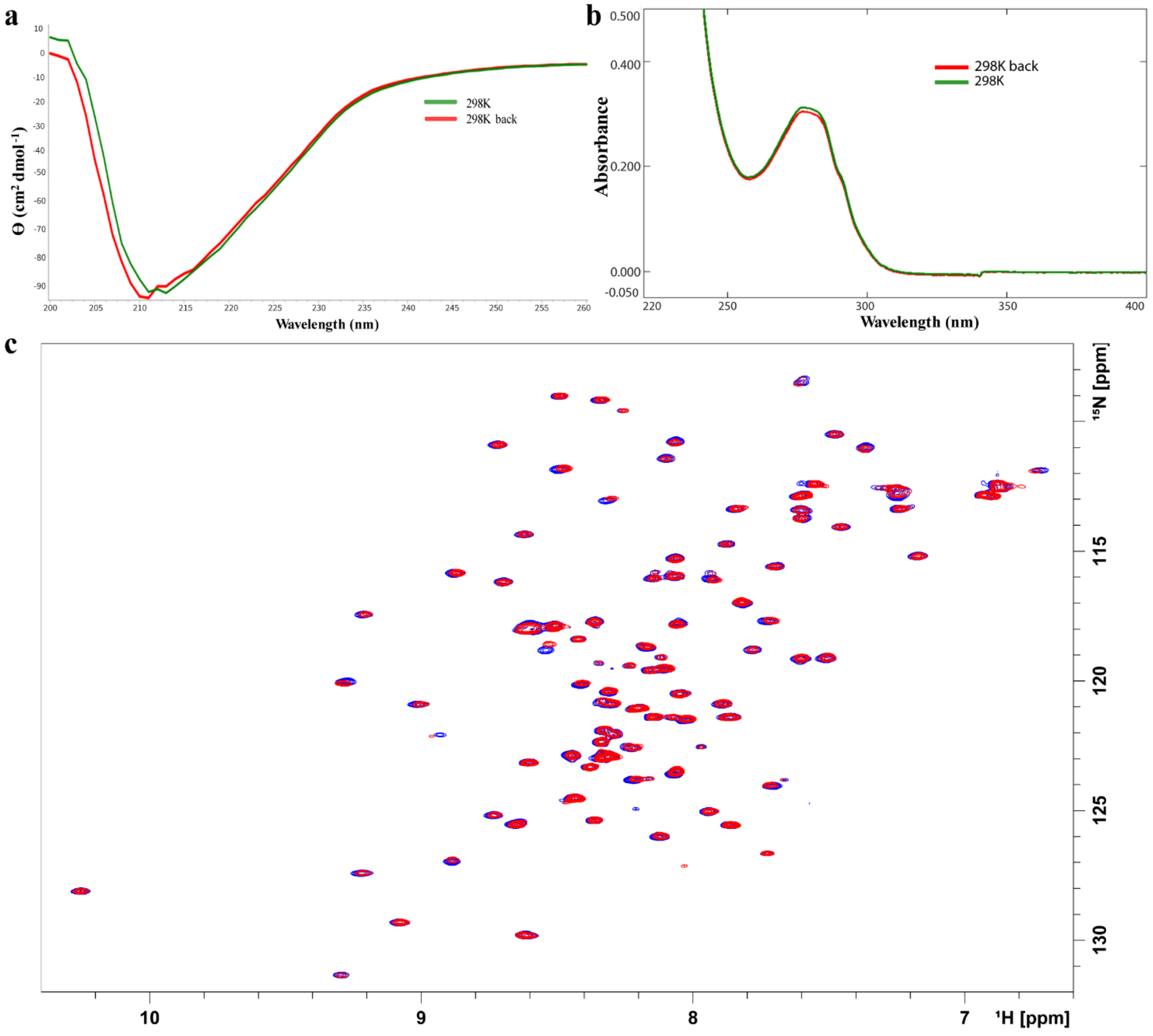

Figure S6. Reversibility of the thermal unfolding of Cd(II)-Ros87. **a)** CD spectra acquired at 298 K before (green) and after heating the sample at 368 K (red). **b)** UV-Vis spectra at 298 K before (green) and after heating the sample at 368K (red). **c)** superposition of the ^{1}H-^{15}N HSQC spectra of Cd(II)-Ros87 at 298K before (blue) and after heating the sample at 343K (red).

© 2020 by the authors. Licensee MDPI, Basel, Switzerland. This article is an open access article distributed under the terms and conditions of the Creative Commons Attribution (CC BY) license (http://creativecommons.org/licenses/by/4.0/).
